# Enhanced Molecular Infrared Spectroscopy Employing Bilayer Graphene Acoustic Plasmon Resonator

**DOI:** 10.3390/bios11110431

**Published:** 2021-10-31

**Authors:** Chunchao Wen, Jie Luo, Wei Xu, Zhihong Zhu, Shiqiao Qin, Jianfa Zhang

**Affiliations:** 1 College of Advanced Interdisciplinary Studies, National University of Defense Technology, Changsha 410073, China; Alexander_Wen@yeah.net (C.W.); jieluo0805@163.com (J.L.); weixu08a@163.com (W.X.); zzhwcx@163.com (Z.Z.); sqqin8@nudt.edu.cn (S.Q.); 2 Hunan Provincial Key Laboratory of Novel Nano-Optoelectronic Information Materials and Devices, Changsha 410073, China

**Keywords:** acoustic graphene plasmons, bilayer graphene, infrared spectroscopy, molecular vibrational fingerprints

## Abstract

Graphene plasmon resonators with the ability to support plasmonic resonances in the infrared region make them a promising platform for plasmon-enhanced spectroscopy techniques. Here we propose a resonant graphene plasmonic system for infrared spectroscopy sensing that consists of continuous graphene and graphene ribbons separated by a nanometric gap. Such a bilayer graphene resonator can support acoustic graphene plasmons (AGPs) that provide ultraconfined electromagnetic fields and strong field enhancement inside the nano-gap. This allows us to selectively enhance the infrared absorption of protein molecules and precisely resolve the molecular structural information by sweeping graphene Fermi energy. Compared to the conventional graphene plasmonic sensors, the proposed bilayer AGP sensor provides better sensitivity and improvement of molecular vibrational fingerprints of nanoscale analyte samples. Our work provides a novel avenue for enhanced infrared spectroscopy sensing with ultrasmall volumes of molecules.

## 1. Introduction

With the ability to probe the vibrational characteristics of inorganic, organic, and polymer compounds samples, infrared spectroscopy possesses the advantages of rapid testing, convenient operation, good repeatability, high sensitivity, and less sample consumption [1,2]. The basic detection principle of infrared spectroscopy is that the position of the vibrational spectra reflects the structural composition of chemical groups, while the intensity of the absorption band is related to the content of chemical groups. The combination of the position and intensity information of the molecular absorption spectra thus allows us to quantitatively and qualitatively identify the structural composition and purity identification of unknown compounds [3,4]. Consequently, infrared spectroscopy has become the most commonly used and indispensable tool in modern biology, chemistry, food safety, and medicine [5,6,7]. However, the sensitivity of traditional molecular infrared spectroscopy is insufficient to address ongoing challenges and emerging applications due to the large mismatch of free-space light (um scale) and testing molecules (<10 nm).

To address this problem, various types of strategies have been proposed to enhance the absorption signal of molecules. Among these strategies, plasmonic enhancement is one of the most effective approaches to improve light–molecule interaction in the mid-infrared range [8,9,10]. Compared to plasmons in traditional noble metals, graphene is two-dimensional (2D) nanomaterials that have been demonstrated to support localized surface plasmons with considerable field confinement in the infrared region, which offer a new class of platform to realize novel electronic and photonic devices in ultracompact sizes [11,12,13]. With the advantages of low propagation loss and actively tunable resonance wavelength in the infrared region, graphene plasmons (GPs) exhibit unique features that are not available in traditional metallic surface plasmons and have been widely used in infrared spectroscopy [14,15,16,17,18,19,20]. Recently, it was demonstrated that graphene plasmons can be tuned and enhanced by double or multi-layer graphene nanostructures [21,22]. Meanwhile, the light confinement and field enhancement can be further boosted when graphene is placed adjacent to a conducting metal surface. In such configuration, an ultra-tightly confined plasmon mode with linear dispersion at small frequencies, so-called acoustic GPs (AGPs), can be excited [23,24,25,26]. The infrared photons are squeezed into extremely confined graphene/nano-gap/metal plasmon cavity down to a sub-nanometric scale and achieve strong interaction between free-space light and atomically thin molecules, which is promising for demonstrating ultimate plasmon confinement limits and ultrasensitive infrared spectroscopy [27,28,29,30,31]. However, the fabrication process of such structure is relatively complex and the metal surface roughness significantly attenuate the excitation far-field signals [30].

In this work, we proposed a resonant graphene plasmonic system to far-field excite AGP mode between a bilayer graphene gap for ultra-sensitive molecular infrared spectroscopy sensing. Here the AGPs are excited in the graphene/nano-gap/graphene plasmon cavity instead of the graphene/nano-gap/metal plasmon cavity. The excitation efficiency of AGPs is improved by placing a reflection mirror below the graphene gratings to form an F–P resonant cavity in the vertical direction. In this bilayer graphene plasmonic biosensor, the tightly confined AGP mode inside the bilayer graphene gap provides strong coupling between ultra-compressed mode-volume plasmon cavity mode and molecular vibrational fingerprints mode, resulting in the significant enhancement of the molecular infrared spectroscopy. For illustration, the protein molecules are selected as the sensing analytes in order to evaluate and analyze the biosensing performance of this bilayer AGPs sensor platform. By adjusting the Fermi energy of graphene via external gate voltage, the AGPs’ resonant wavelength can be actively tuned over the infrared region, which enables us to sensitively detect the absorption band of ultra-thin target molecules.

## 2. Materials and Methods

**Figure 1:**
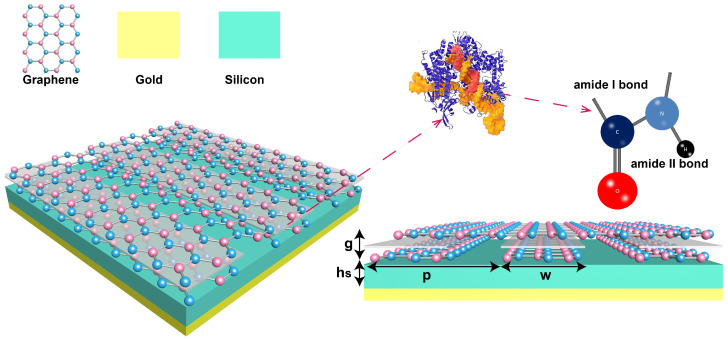
Schematic of the graphene plasmonic system including from top to bottom: continuous graphene, nano-gap and graphene ribbons, dielectric spacer layer, and metal mirror substrate. The protein sensing analytes are placed inside the nano-gap. THe peptide bond, a basic chemical bond of the polypeptide chain, includes the C=O bond (amide I) and C–N bond (amide II).

The ultra-compressed mode-volumes graphene plasmon cavity are formed by graphene/ nano-gap/graphene van der Waals heterostructure with a period of *p* = 82 nm, a width of graphene grating *w* = 42 nm, and a thickness of nanogap *g* = 3 nm as shown in Figure 1. The continuous graphene plasmonic cavity array are placed over a dielectric layer (silicon) with a thickness $h_{s}$ = 410 nm and gold mirror substrate. The simulation is perfromed by Comsol Mutiphysics software. Graphene is regarded as transition boundary conditions and characterized by its surface conductance σ(ω) described by Drude-like formula [32,33]

(1) $$\sigma \left(\omega \right)=\frac{e^{2}E_{F}}{\pi \hbar^{2}}\frac{i}{\omega +i{\tau_{s}}^{-1}}$$

where $E_{F}$ is the Fermi energy of graphene, *e* is the elementary charge, *ℏ* is the reduced Planck constant, τ is the electron relaxation time calculated from $\tau_{s}=\mu E_{F}/e{V_{F}}^{2}$, $V_{F}=c/300$ is the Fermi velocity, *c* is the speed of light in vacuum, and μ is the carrier mobility of graphene where μ = 10,000 cm${}^{2}$(vs)${}^{-1}$. The Fermi energy of graphene is 0.6 eV if not specially mentioned below, which can be tuned by means of chemical doping or electric doping. The metal selected is gold, whose optical permittivity is described by the Drude–Lorenz model

(2) $$\varepsilon_{m}=\varepsilon_{\infty }-\frac{{\omega_{p}}^{2}}{\omega^{2}+i\tau \omega }$$

where $\omega_{p}=1.37\times 10^{16}$ rad/s and $\tau =8.17\times 10^{13}$ rad/s. The period boundary condition is employed in the *x*-direction and that in the *y*-direction is infinitely. The refraction index of silicon spacer layer is $n_{s}$ = 3.4 and the relative permittivity of nano-gap before replacing by testing protein analytes is assumed to be $\varepsilon_{p}$ = 2.08.

## 3. Results and Discussion

### 3.1. Excitation of Bilayer Acoustic Graphene Plasmons

We first investigate the excitation of graphene acoustic plasmons in the proposed bilayer plasmonic system. Under the illumination of transverse magnetic (TM) waves, graphene plasmons can be excited with the assistance of the patterned graphene nanoribbons. According to the F–P resonant model, the resonance condition of such graphene plasmonic modes can be given as following equation:

(3) $$Re\left(k_{spp}\right)\left(p-w\right)+Re\left(k_{aspp}\right)w+\phi^{{}^{\prime }}+\phi^{{}^{\prime \prime }}=2m\pi$$

Here, $k_{spp}$ denotes the momentum of the traditional GPs excited in the single-layer graphene region, and $k_{aspp}$ denotes the momentum of the AGPs excited in the bilayer graphene region, respectively. An integer *m* represents the order of the resonance, Re means taking the real part of the complex quantity, and $\phi^{{}^{\prime }}$ and $\phi^{{}^{\prime \prime }}$ are the phase shift of grating edge reflection, which are zero in this bilayer system [30].

**Figure 2:**
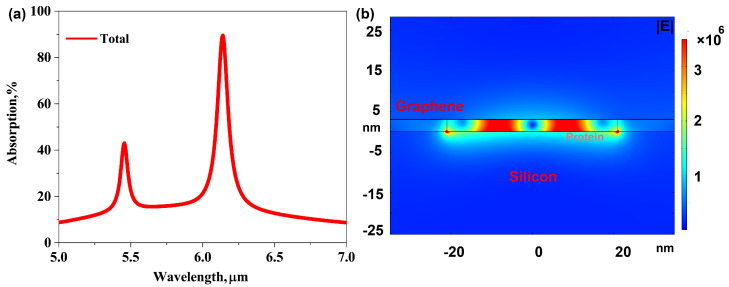
(**a**) The absorption spectrum of bilayer AGPs system for TM waves. (**b**) Electric mode distribution of the bilayer AGP system at wavelength $\lambda_{0}$ = 6.14 μm. The AGP mode is excited and optical field is confined tightly inside nano-gap of bilayer graphene plasmon cavity.

In order to improve the conversion efficiency of graphene acoustic plasmon, the dielectric spacer layer and gold mirror are further introduced to form an F–P resonance cavity [30,34] to enhance the absorption of graphene plasmons. The total absorption spectrum (see red line) of the proposed graphene plasmonic system is shown in Figure 2a. A resonant peak can be observed located around at wavelength $\lambda_{0}$ = 6.14 μm, which can be ascribed to the excitation of bilayer graphene gap plasmons mode in nano-gap, the so-called AGP mode. The corresponding spatial distribution of the electric field |E| is plotted in Figure 2b. The figure shows that the optical field is tightly confined within the bilayer graphene gap. The ultra-compressed mode-volume equals approximately to the volume of the nano-gap inside the graphene plasmonic cavity. Such highly confined plasmonic cavity allows us to realize out-of-plane confinement of propagating plasmons down to a sub-nanometric thick layer at the atomic scale [27] (here, ∼$\lambda_{0}$/4000), which significantly boosts interaction between incident light and molecules.

### 3.2. Protein Molecular Vibrational Fingerprints

**Figure 3:**
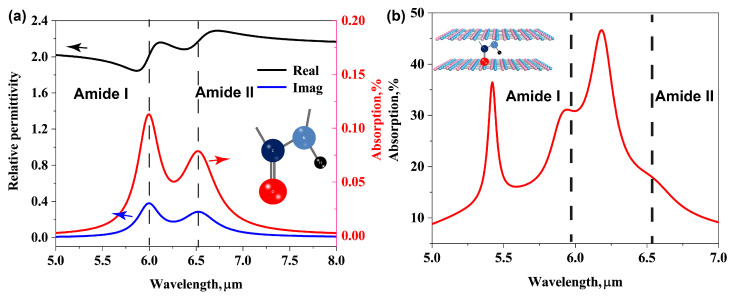
(**a**) Real and imaginary parts of the relative permittivity of this protein are shown as black and blue curves, respectively. The red line show the absorption of 3nm thickness protein molecules coated above losses dielectric substrate (refraction index n = 1.4 and thickness h = 200 nm). (**b**) The absorption spectrum of the bilayer AGP system after placing protein molecules inside plasmon cavity.

The peptide bond -CO-NH- in proteins is formed by the dehydration condensation reaction of a α-amino of one molecule and α-carboxyl of another molecule. Two absorption bands in spectra are primarily associated with the C=O bond and N–H bond in the amide functional group (see Figure 1). The relative permittivity $\varepsilon_{\omega }$ of protein sensing analytes used in this work is described by Drude–Lorentz like formula Equation (4) [14].

(4) $$\varepsilon_{\omega }=\varepsilon_{\infty }+\frac{W_{1}}{\omega_{p1}^{2}-\omega^{2}-i\tau_{1}}+\frac{W_{2}}{\omega_{p2}^{2}-\omega^{2}-i\tau_{2}}$$

where the values of different terms $\varepsilon_{\infty }$ = 2.08, $\omega_{p1}$ = 1668 cm${}^{-1}$, $W_{1}$ = 213 cm${}^{-1}$, $\tau_{1}$ = 78 cm${}^{-1}$ and $\omega_{p2}$ = 1532 cm${}^{-1}$, $W_{2}$ = 200 cm${}^{-1}$, $\tau_{2}$ = 101 cm${}^{-1}$. The real and imaginary parts of complex permittivity are plotted in Figure 3a. Besides, the absorption spectra of *g* = 3 nm thickness protein coating over a lossless silicon dioxide substrate is plotted by red line in this picture, and the maximum value of absorption peak are 0.11% and 0.078%, indicating the positions (dash line) of the amide I band (5.99 μm) and the amide II band (6.53 μm), respectively.

### 3.3. Enhanced Infrared Absorption Spectroscopy

**Figure 4:**
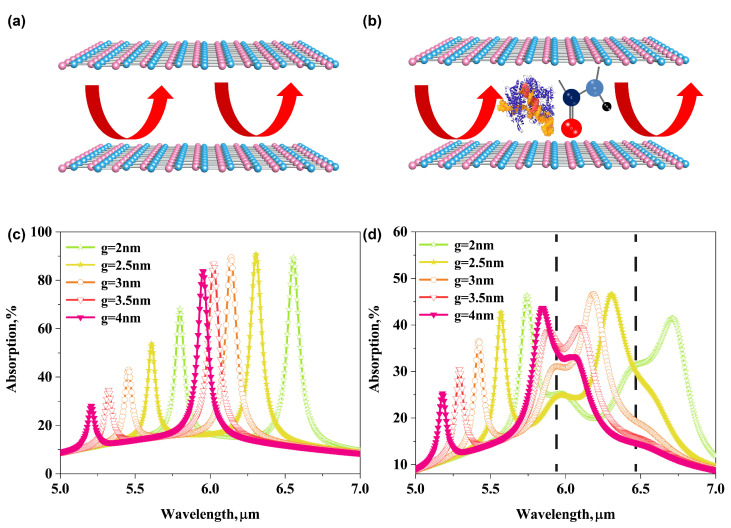
(**a**) Before coating protein molecules. (**b**) After coating proteins molecules, and the coupling mechanism of graphene-dielectric-graphene plasmon cavity mode and molecular vibrational fingerprints mode. (**c**) Extinction spectra of the bilayer AGPs system at varying the thickness of nano-gap. (**d**) Extinction spectra of the bilayer AGPs system with 3 nm protein inside nano-gap at varying the thickness of nano-gap. The dashed lines in this picture show the positions of vibrational band of protein molecules.

In order to corroborate the infrared absorption spectroscopy enhancement performance of the bilayer graphene plasmonic system, we now turn to compare the optical response of the plasmonic system with a dielectric layer and protein molecular layer embedded in the nano-gap, as shown in Figure 4a,b. The figures briefly describe the coupling mechanism between a graphene plasmonic mode and a molecular vibrational mode. According to the mode-coupling theory [35], the infrared absorption spectroscopy of sub-nanometric volumes protein molecules can be effectively enhanced by the strong coupling between the graphene plasmonic mode and the molecular vibrational mode due to the strong plasmonic field confinement squeezed inside the bilayer graphene plasmon cavity.

To prove the enhancement mechanism mentioned above, the extinction spectra of the two bilayer graphene plasmonic systems with varying thickness of nano-gap g are calculated and compared in Figure 4c,d. For the system with the dielectric layer, it is clear that the plasmonic resonant peak red-shifts from 5.95 μm to 6.55 μm as *g* decreases from 4 nm to 2 nm with a step of 0.5 nm, which covers the absorption band of amide I and amid II, as shown in Figure 4c. The shifts of the plasmonic resonant peak can be understood by the fact that the plasmonic wave vector $k_{spp}$ or effective mode refraction index (EMRI = $k_{spp}$/$k_{0}$, $k_{0}$ denotes the wave vector in vacuum) of AGP mode in a graphene-insulator-metal waveguide is decided by the nano-gap thickness g and refraction index of nano-gap material via a simple proportional relationship of $1/\sqrt{g}$ [30,36,37]. Especially when the *g* is small enough, the shifts of the resonant peak are more sensitive to the value of *g*. When the dielectric layer is replaced by a protein molecular layer, the absorption spectra of the graphene plasmonic system are dramatically affected by the amide bands and molecular fingerprints. The peaks and dips of the sweeping spectrum coincide well with the positions of the amide band. The absorption of the protein molecules is greatly enhanced by the AGP mode compared to the intrinsic absorption shown in the red line in Figure 3a, which allows us easily recognize the molecular vibrational modes from Figure 4d by comparing them to plasmonic spectra in Figure 4c. The above results indicate that the tightly compressed mode volume enabled by AGP mode in the proposed bilayer graphene plasmonic system is promising for achieving strong overlap and coupling of plasmons mode with molecular vibrational fingerprints mode.

### 3.4. Detection of Molecular Fingerprints by Tunable Graphene Acoustic Plasmons

**Figure 5:**
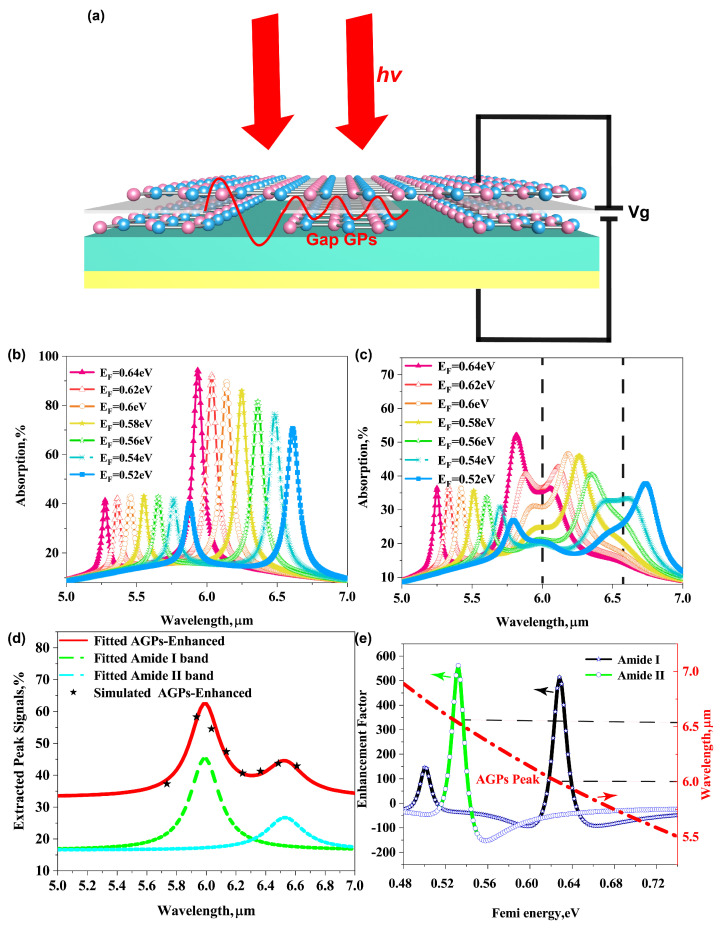
(**a**) Conceptual view of the bilayer AGPs mid-IR tunable biosensor. (**b**) Extinction spectra of the bilayer AGPs biosensor by varying $E_{F}$ of doping graphene. (**c**) Extinction spectra of the biosensor with 3 nm protein in nano-gap by varying $E_{F}$. The dashed lines show the positions of the absorption band of molecules. (**d**) Lorentz fitted molecular absorption enhanced by AGPs. (**e**) The calculation for enhancement of the molecular absorption at amide I and II bands at varying $E_{F}$.

The graphene plasmon mode possesses particularly the advantage of ultra-broad and fast tunability by tuning the Fermi energy via electrical doping [3,38]. Thus, the molecular vibrational fingerprints of protein sensing analytes can be sensitively detected by gate voltage controlling. A schematic of a tunable bilayer AGP mid-infrared biosensor is shown in Figure 5a. The AGPs are excited inside the graphene gap and tuned by grating voltage $V_{g}$. Protein sensing is achieved by detecting the spectral shift of plasmon resonance accompanied by narrow dips corresponding to the molecular vibration bands of the protein. The absorption spectra of the bilayer acoustic graphene plasmonic system with varying Fermi energy $E_{F}$ are shown in Figure 5b. It is noted that with $E_{F}$ decreasing from 0.64 eV to 0.52 eV, the first-order AGP peak red-shifts from around 5.935 μm (absorption 94.33%) to 6.61 μm (absorption 70.63%). After coating the 3 nm thick protein molecule layer, the absorption dips corresponding to the amide band are observed in Fermi energy sweeping spectra, as shown in Figure 5c. The strength of peaks and dips are decided by the shifts of graphene plasmons resonant wavelength compared to the positions of the amide band.

By tuning the Fermi energy of doping graphene, the dips become gradually more intense with increasing spectral overlap between the plasmon resonant peak and protein molecular vibrational bands. The absorption peak difference (pentagram point) feature of protein vibrational modes after plasmonic enhancement with varying graphene Fermi energy is further extracted and plotted as a function of wavelength in Figure 5d. Then the peak difference extinction was fitted using two-peak Lorentz line shape to obtain the absorption bands of protein molecules enhanced by bilayer AGPs. The peaks were selected at around 5.99 μm and 6.53 μm. The fitting function is shown in Appendix A Equation (A1) and the fitting curves are plotted in Figure 5d. The fitting absorption spectrum of protein molecules shows good agreement with the simulation results. Moreover, the absorption intensity is significantly enhanced compared to the intrinsic absorption of protein molecules shown in Figure 3a. Quantitatively, the enhancement factor of protein molecule absorption for amide I and II as a function of Fermi energy are plotted in Figure 5e. For illustration, a maximum enhancement factor of 510 can be achieved at $E_{F}$ = 0.628 eV (extracted difference of peak signals 56.62% compared to absorption 0.11%) for the amide I band and 560 at $E_{F}$ = 0.532 eV (extracted difference of peak signals 43.81% compared to intrinsic absorption 0.078%) for the amide II band, respectively, where the plasmon peaks rightly move to the positions of the molecular absorption band. Such high enhancement factors are approximately one order of magnitude larger than that of graphene nano-ribbons biosensor, indicating the excellent sensing performance of the designed bilayer AGP sensors [39].

### 3.5. Comparison between Bilayer AGP Biosensor and GP Biosnesor

**Figure 6:**
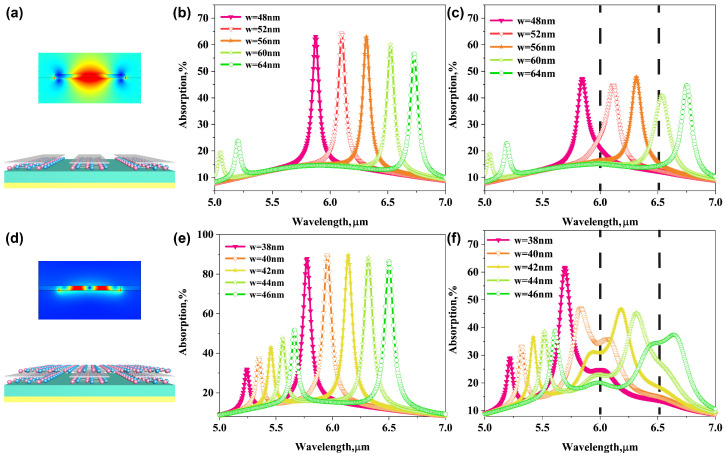
Electric filed distribution of (**a**) conventional GP biosensor and (**d**) bilayer AGP mid-IR biosensor. (**b**) Extinction spectra of the GP system by varying width *w* of graphene ribbons. (**c**) Extinction spectra of the GP system with 3 nm protein in nano-gap by varying width *w* of graphene ribbons. (**e**) Extinction spectra of the bilayer AGP system by varying width *w* of graphene ribbons. (**f**) Extinction spectra of the bilayer AGP system with 3nm protein in nano-gap by varying width *w* of graphene ribbons.

To illustrate the advantage of tunable bilayer AGP biosensor, we finally compare the enhancement performance of the proposed bilayer AGP system to that of single-layer GP biosensor by removing top continuous graphene sheet, as shown in Figure 6a,d. Their corresponding near-field distributions around the graphene ribbons are also provided in the insets. It is clearly shown the electromagnetic field of AGPs exhibits much higher confinement that is tightly confined in the nano-metric gap between the bilayer graphene, resulting in a much larger spatial overlap between the plasmonic field and protein molecules compared to conventional GPs mode excited in solely graphene nanoribbons. Specifically, most part of the mode energy is confined within a 3 nm nano-gap for the AGP biosensor, while the same percentage of mode energy in GP biosensor is spread over a distance of 15 nm away from the graphene surface [14]. This allows us in principle to achieve stronger interaction of free-space light and protein molecules.

The extinction spectra of the GP sensor and AGP biosensor with varying widths of graphene ribbons are shown in Figure 6b,e, respectively. For the GP biosensor, the resonant peak red-shifts from about 5.875 μm to 6.725 μm as the ribbon width increase from 48 nm to 64 nm with increment of 4 nm (Figure 6b). While for AGP biosensor, the resonant peak red-shifts from about 5.775 μm to 6.5 μm as the ribbon width increase from 38 nm to 46 nm with increment of 2 nm (Figure 6e). It is clear that the shift of plasmon peak of bilayer AGP biosensor is more sensitive to the change of ribbon width value because the plasmonic wave vector $k_{aspp}$ or EMRI in graphene-insulator-graphene waveguide region is much larger than that in single-layer graphene at same mid-IR frequency. After coating the 3 nm protein molecular layer, we nearly cannot observe the vibrational dips of protein molecules from the extinction spectra of conventional GP sensor, and thus it is hard for us to recognize the analyte coating on the graphene surface, as shown in Figure 6c. In contrast, obvious dips associated with the amide bands can be observed from the absorption spectra of AGPs sensor, as shown in and Figure 6f. This result confirms that the bilayer AGPs provide stronger light-biomolecules interactions compared to the conventional GP sensor, leading to the better sensitivity and improvement of molecular vibrational fingerprints spectral resolution.

### 3.6. Discussions about the Fabrication of the Proposed Device

Our proposed structure can be fabricated with a similar process as previous literature [14,30,34]. The main difference of fabrication steps for our AGP biosensor and GP ribbon sensor [14,34] is final continuous graphene sheet transferring. Firstly, the gold reflector can be defined on oxide substrate by photolithography and lift-off. Secondly, the dielectric spacer layer with different thicknesses can be deposited on the metal reflector by magnetron sputtering or electron beam deposition. Thirdly, a monolayer graphene sheet can be wet-transferred on the top of the optical dielectric spacer and patterned into nano-ribbon arrays with electron beam lithography. For AGP enhanced molecular infrared spectroscopy, a solution of protein sensing analytes can be prepared according to previous work [34] and spin-coated on the graphene nano-ribbons before transferring the top graphene. The infrared spectra of the device can be acquired with a Fourier-transform infrared spectrometer.

## 4. Conclusions

In conclusion, we have suggested a method of probing vibrational fingerprints of extremely ultra-small volume molecules with a bilayer graphene acoustic plasmons biosensor, which is formed by continuous ultra-compressed mode-volume graphene-insulator-graphene plasmon cavity. Compared to graphene-gap-metal AGP structures, the proposed design is expected to solve the difficulty of metal unevenness or surface roughness which significantly attenuates far-field signals and requires a complex fabrication process to realize ultraflat metal surfaces. Furthermore, we have found that strong absorption and tight electromagnetic field confinement are realized in mid-infrared frequency for such a biosensor. The acoustic plasmons modes are squeezed into the nano-metric gap by far-field excitation with high efficiency at normal incidence. By placing protein molecules inside bilayer graphene nano-gap, the molecular fingerprints can be frequency-selectively enhanced by extreme plasmon overlap. The extinction spectra and the absorption band of thin protein molecules can be precisely resolved by sweeping the graphene Fermi energy and extracting plasmon-enhanced excitation peak signals. The maximum enhancement factor can be up to 510 for the amide I band and 560 for the amide II band of enhanced molecular absorption IR spectroscopy. We further demonstrate that the sensitivity of the AGP biosensor is much higher than that of the conventional GP biosensors due to highly confined plasmon mode overlap (<3 nm) and ultra-strong interaction of light and biomolecules. Our proposal provides a new platform to study nanoscale light-matter interactions and ultrasensitive infrared spectroscopy for single-molecule optics, extreme biosensing [40,41], and even quantum plasmonics [42,43].

## Data Availability

Relevant data is included in the manuscript.

## Appendix A

The peak difference data of extinction spectra simulated by sweeping Fermi energy in Mutiphyscis Software was fitted using two-peaks Lorentz line shape to obtain the absorption bands of protein molecules enhanced by bilayer AGPs. The double-peak Lorentz absorption function is as follows:

(A1) $$y=y_{0}+\left(2A/\pi \right)\frac{w}{4{\left(\lambda -\lambda_{c}\right)}^{2}+w^{2}}$$

**Table A1:** The double-peak Lorentz absorption function parameters of extinction spectra enhanced by bilayer AGPs.

Peak	$y_{0}$	A	$\lambda_{c}$	w
Amide I	0.165	0.109	5.990	0.241
Amide II	0.165	0.049	6.530	0.305
